# Chronic Inflammatory Tunica Vaginalis Masses With Haemorrhagic Hydrocoele Fluid: A Rare Finding in a Patient With Vaginal Hydrocoele

**DOI:** 10.7759/cureus.94247

**Published:** 2025-10-09

**Authors:** Paul E Ngwu, Ifeanyichukwu E Ihedoro, Chibueze P Ohiarah

**Affiliations:** 1 Urology, Federal Medical Center, Umuahia, Umuahia, NGA; 2 Urology, Eleos Specialist Hospital, Umuahia, NGA; 3 Urology, North Cumbria Integrated Care NHS Foundation Trust, Carlisle, GBR; 4 Surgery, Federal Medical Center, Umuahia, Umuahia, NGA

**Keywords:** haemorrhagic hydrocoele, inflammatory, paratesticular masses, tumours, tunica vaginalis

## Abstract

Tunica vaginalis masses are a part of the paratesticular masses, which are rare. The combination of the finding of tunica vaginalis masses with haemorrhagic hydrocele fluid will make for a high index of suspicion of a mitotic lesion. Most reported cases of tunica vaginalis masses are mesotheliomas, most of which present as painless scrotal swellings with hydrocele.

Our patient is a 58-year-old man who presented with a painless swelling involving the left hemiscrotum, in which the histopathological findings following surgery turned out to be chronic inflammatory tunica vaginalis masses.

Chronic Inflammatory tunica vaginalis masses, even though rare, should be borne in mind when thinking of possible differential diagnosis in a patient with painless scrotal swelling.

## Introduction

Tunica vaginalis masses are a type of paratesticular masses, which are rare. The combination of the finding of tunica vaginalis masses with haemorrhagic hydrocoele fluid will make for a high index of suspicion of a mitotic lesion. Most reported cases of tunica vaginalis masses are mesotheliomas [1,2], most of which present as painless scrotal swellings with hydrocoele. Tumours and tumour-like lesions of the tunica vaginalis are rare; benign tumours of the tunica vaginalis account for about 70% of these tumours. A definitive diagnosis will mostly be made through histological analysis of these tumours [3,4]. The presence of haemorrhagic hydrocoele in addition to the other intraoperative findings puts malignant lesions high up in the list of differentials for the index patient.

## Case presentation

**Figure 1:**
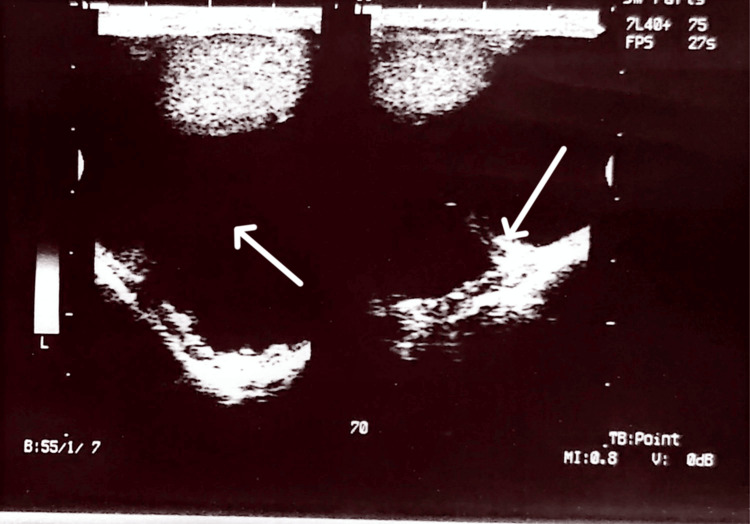
Ultrasound image with arrows pointing to the hydrocele fluid between the visceral and parietal layers of the tunica vaginalis in the first image and a probable mass in the second image

A 58-year-old man presented with a painless left hemiscrotal swelling that was noticed four years before presentation. The swelling was gradually increasing in size over the years. There was no history of trauma, no lower urinary tract symptoms (LUTS), no previous groin or scrotal surgeries and no history of previous radiation therapy or exposure to asbestos in the patient. Physical examination revealed a fluctuant swelling that was transilluminable, suggestive of vaginal hydrocele, which was confirmed by scrotal ultrasound scan, as shown in Figure 1.

**Figure 2:**
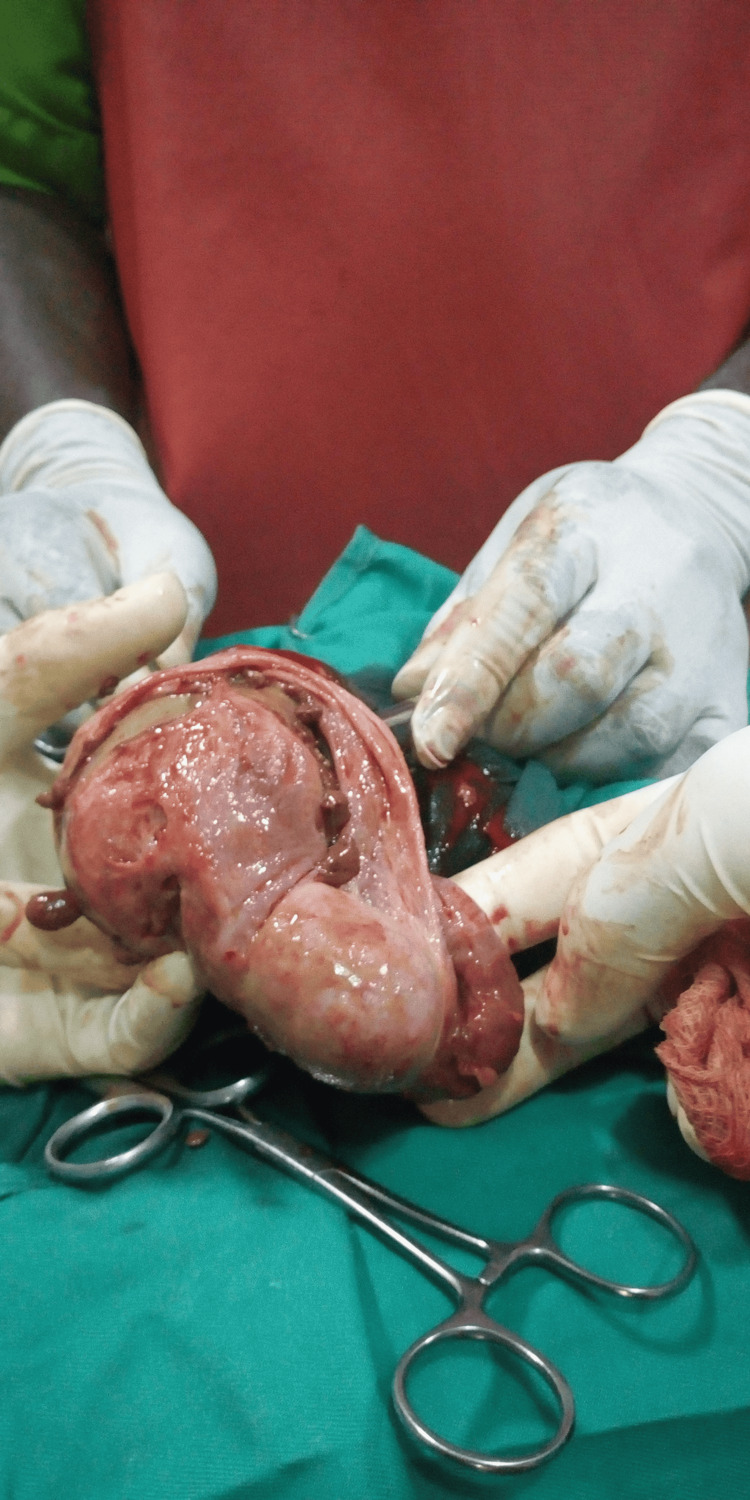
Showing the various masses on the tunica vaginalis as seen intraoperatively

The patient was worked up and booked for hydrocoelectomy. Under spinal anaesthesia, a left hemiscrotal transverse incision was made and deepened to the parietal layer of the tunica vaginalis. About 250 mL of haemorrhagic fluid was drained on incising the tunica vaginalis. Two free-floating greyish soft masses, the largest of which was about 2.5cm in diameter, were seen within the fluid with pedunculated masses of varying sizes on the parietal layer of the tunica vaginalis, as shown in Figure 2.

**Figure 3:**
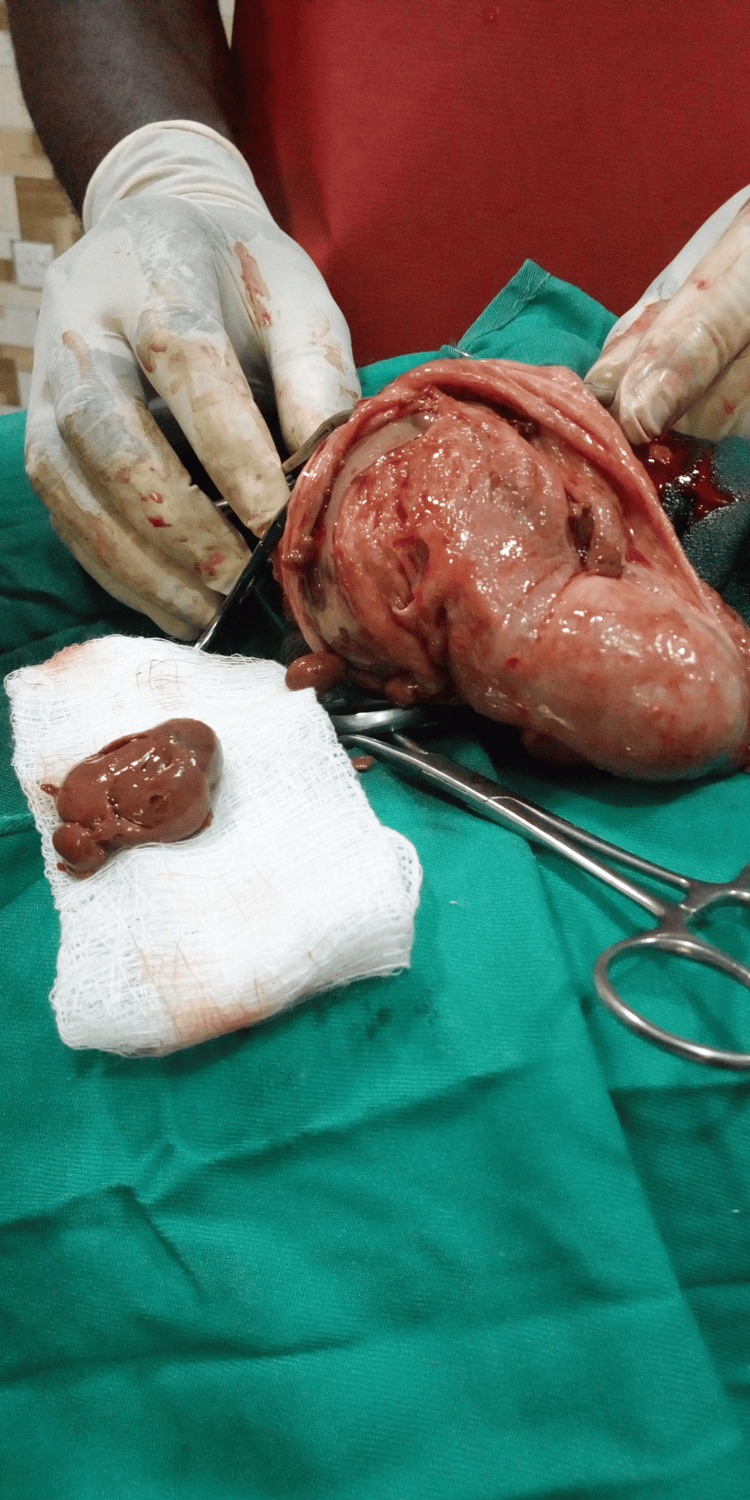
Showing one of the masses post-hydrocelectomy

Hydrocoelectomy (modified Jaboulay’s procedure ) was done with excision of the part of the sac (tunica vaginalis) with obvious masses (one of the post op masses shown in Figure 3 below). Bleeding from the edges of the resection was controlled with the aid of a combination of diathermy and suturing using Vicryl 2/0.

**Figure 4:**
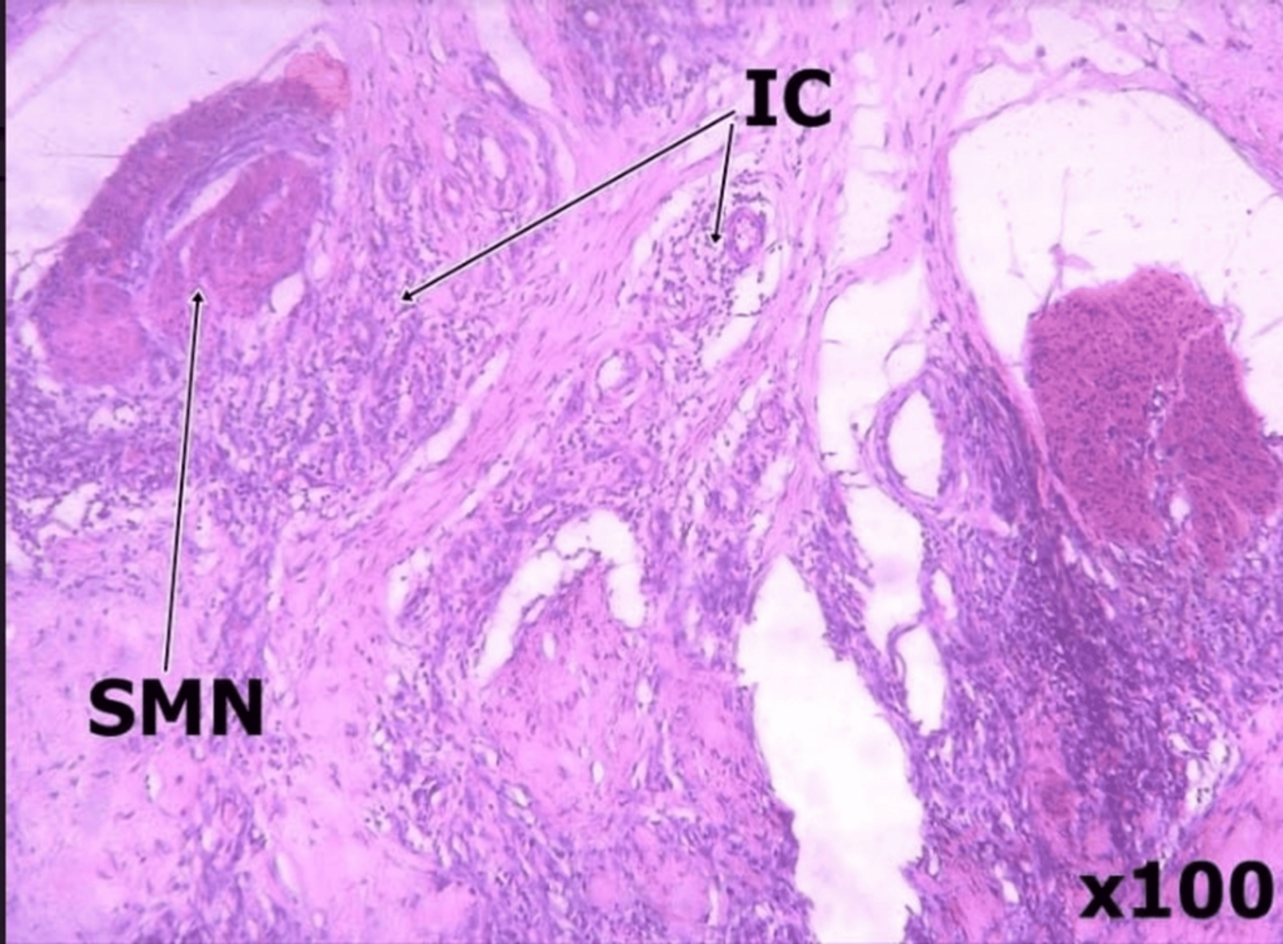
Tissue histologic section shows an oedematous wall made of fibrocollagenous tissue interspersed by small nodules of smooth muscle fascicles (SMN) and mild to moderate chronic inflammatory exudate (IC)

**Figure 5:**
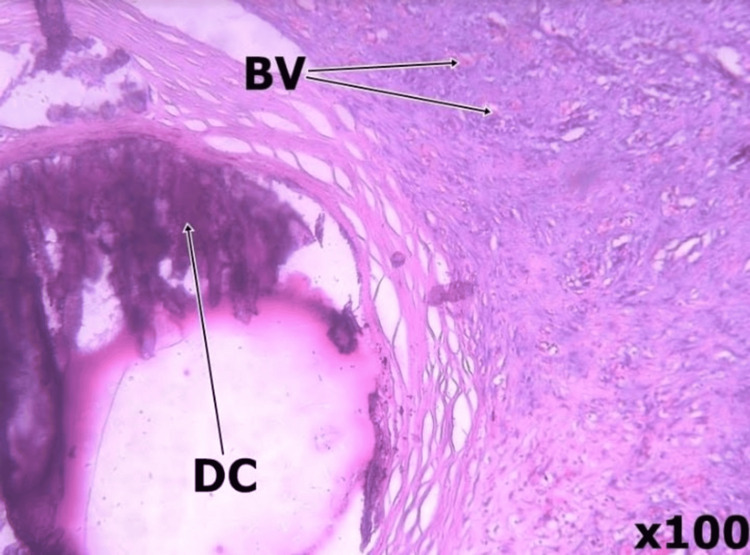
Tissue histologic section showing proliferating small caliber blood vessels (BV), foci of dystrophic calcifications (DC) and blood clots with no atypia.

Histological analysis of the specimen showed chronic inflammation with no evidence of malignancy, as shown in Figures 4, 5 below.

The patient was discharged on the same day of surgery, and post-operative visits have been uneventful for the past year. 

## Discussion

Paratesticular masses, of which tunica vaginalis masses are a component, account for approximately 30% of all scrotal masses, and 70% of these masses are benign [3,4]. The paratesticular region includes the spermatic cord contents, testicular tunics, epididymis, and vestigial remnants [5]. Histologically, this area is composed of a variety of epithelial, mesothelial, and mesenchymal elements.

Generally, tunica vaginalis masses are either benign or malignant. When a patient presents with a hydrocoele that contains hemorrhagic fluid, the possibility of malignancy, such as malignant mesothelioma of the tunica vaginalis, paratesticular rhabdomyosarcoma, and testicular tumour, should be considered. Malignant mesothelioma of the tunica vaginalis is a rare and highly aggressive tumour, often accompanied by a hydrocoele [6-9]. Histologically, the benign differential diagnosis of this tumour includes fibrous pseudotumour, solitary fibrous tumour, leiomyoma, neurofibroma, fibroma of the tunics, and fibromatosis [10].

The obvious preoperative findings in our patient were a painless scrotal swelling that was fluctuant with the discovery of haemorrhagic hydrocoele, a thickened tunica vaginalis, as well as masses during exploration. This made us have a high index of suspicion of malignancy.

The scrotal ultrasound scan done for this patient pointed to vaginal hydrocele. Ultrasound can be useful in distinguishing a paratesticular mass from an intratesticular mass [9]. Unfortunately, there are no ultrasound features that can definitively differentiate the above lesion from malignant testicular masses [11].

Our patient had a modified Jaboulays procedure, keeping in view radical orchidectomy if histology shows a malignant lesion.

## Conclusions

This case report highlights the challenges in preoperatively making a diagnosis of paratesticular masses, particularly those involving the tunica vaginalis. The presence of a painless scrotal swelling, which is the most common presentation, combined with hemorrhagic hydrocele fluid and masses found intra-operatively, creates a high suspicion of malignancy. In the index case, the definitive diagnosis came from a histological analysis, which revealed the masses were chronic inflammatory tunica vaginalis masses with no evidence of malignancy. This outcome underscores the critical role of histological analysis in differentiating between benign and malignant lesions, especially when clinical and radiological findings are suggestive of malignancy.

This case is reported due to its rarity and serves as a reminder that chronic inflammatory tunica vaginalis masses, although uncommon, should be considered in the differential diagnosis for patients presenting with a painless scrotal swelling. The findings from this patient's case underscore the difficulty in accurately diagnosing these types of masses pre-operatively. Therefore, when a similar case scenario presents, this benign tumor should be borne in mind as a possibility, despite the high index of suspicion for a more serious condition. Ultimately, the case demonstrates that while certain findings may point toward a malignant lesion, a definitive diagnosis relies on post-operative histological analysis.

## References

[1] Zhang N, Fu N, Peng S, Luo X (2017). Malignant mesothelioma of the tunica vaginalis testis: a case report and literature review. Mol Clin Oncol.

[2] Chen JL, Hsu YH (2009). Malignant mesothelioma of the tunica vaginalis testis: a case report and literature review. Kaohsiung J Med Sci.

[3] Unlü Y, Huq GE, Ozyalvaçli G (2015). Paratesticular sarcomas: a report of seven cases. Oncol Lett.

[4] Barazani Y, Tareen B (2012). Rare case of paratesticular solitary fibrous tumour (lipomatous hemangiopericytoma). Can Urol Assoc J.

[5] Makhdoomi R, Bashir H, Muanfat M, Baba KM (2012). Primary paratesticular carcinoid in a 70-year-old male. Cent European J Urol.

[6] Chekol SS, Sun CC (2012). Malignant mesothelioma of the tunica vaginalis testis: diagnostic studies and differential diagnosis. Arch Pathol Lab Med.

[7] Barbera V, Rubino M (1957). Papillary mesothelioma of the tunica vaginalis. Cancer.

[8] de Sá Barrêto Callou Peixoto M, Bernardo Soares MK, Libânio BB, Albuquerque KS, Bacchi CE (2022). Malignant mesothelioma of the tunica vaginalis testis: a rare cause of hydrocele. Urol Case Rep.

[9] Stella S, Ceresoli GL, Dallari B (2024). Mesothelioma of the tunica vaginalis testis: diagnostic and therapeutic management. A comprehensive review, 1982-2024. Cancers (Basel).

[10] Mutreja D, Murali M, Arya A (2013). Pseudotumors of paratesticular region mimicking malignancy. Arch Int Surg.

[11] Tammela TL, Karttunen TJ, Mäkäräinen HP (1991). Intrascrotal adenomatoid tumors. J Urol.

